# Periodicals and Pamphlets

**Published:** 1873-08

**Authors:** 


Periodicals and Pamphlets.—The American Agriculturist published by Orange, Judd & Co., of New York, is a well known, very able and enterprising publication. It is furnished to the public at a minimum rate and is crowded with valuable articles. Among its excellent features we notice the monthly *expose* it presents of the medical humbugs that are having their run. Messrs. Judd & Co. also publish the Hearth and Home, an illustrated family paper issued weekly.

As a premium to subscribers they present with the "Agriculturist" a Chromo entitled "Mischief Brewing," and with "Hearth and Home" one called "The Strawberry Girl." We have to acknowledge the receipt of these Chromos, and can honestly pronounce them to be excellent. They offer these two papers with both Chromos at the low price of $4.75 for the year.

The first number of the New Orleans Medical and Surgical Journal is announced to appear July 1st. The Journal will be issued on every alternate month, and will contain one hundred and fifty pages. The Journal is to be under the editorial management of Dr. S. M. Bemis. We extend to the New Journal our best wishes for its success.——The Globe is the name of a new monthly magazine published in this city by Mr. E. L. Cornwell. Each number consists of sixteen pages well filled with instructive reading matter. It numbers among its contributes some of the best writers of the city and vicinity, and well deserves the support of our citizens.

